# Genome-wide association and RNA-seq analyses identify loci for pod orientation in rapeseed (*Brassica napus*)

**DOI:** 10.3389/fpls.2022.1097534

**Published:** 2023-01-13

**Authors:** Yuting Yang, Wenxiang Wang, Qiong Hu, Harsh Raman, Jia Liu

**Affiliations:** ^1^ Oil Crops Research Institute, Chinese Academy of Agricultural Sciences, Wuhan, Hubei, China; ^2^ Shenzhen Graduate School, Chinese Academy of Agricultural Sciences, Shenzhen, Guangdong, China; ^3^ Key Laboratory of Biology and Genetic Improvement of Oil Crops, Ministry of Agriculture and Rural Affairs, Wuhan, China; ^4^ New South Wales (NSW) Department of Primary Industries, Wagga Wagga Agricultural Institute, Wagga Wagga, NSW, Australia

**Keywords:** rapeseed, pod orientation, GWAS, RNA-seq, candidate genes

## Abstract

Spatial distribution and orientation of pods on the main raceme (stem) and branches could affect rapeseed yield. However, genomic regions underlying the pod orientation were not described in *Brassica* species. Here, we determined the extent of genetic variation in pod orientation, described as the angles of pedicel on raceme (APR) and angles of the pod on pedicel (APP) among 136 rapeseed accessions grown across three environments of the upper, middle and lower Yangtze River in China. The APR ranged from 59° to 109°, while the APP varied from 142° to 178°. Statistical analysis showed that phenotypic variation was due to genotypic (G) and environmental (E) effects. Using the genome-wide association analysis (GWAS) approach, two QTLs for APR (qBnAPR.A02 and qBnAPR.C02) and two for APP (qBnAPP.A05 and qBnAPP.C05), having minor to moderate allelic effects (4.30% to 19.47%) were identified. RNA-seq analysis revealed 606 differentially expressed genes (DEGs) in two rapeseed accessions representing the extreme phenotypes for pod orientation and different alleles at the QTLs of APR. Three DEGs (*BnLAZY4.A02*, *BnSAUR32.A02*, and *BnSAUR32.C02*) were identified as the most likely candidates responsible for variation in pod orientation (APR). This study elucidates the genomic regions and putative candidate genes underlying pod orientation in *B. napus*.

## 1 Introduction

Rapeseed (*Brassica napus* L.) is the third largest source of edible vegetable oil, mainly grown for human consumption in various parts of the world, especially in Australia, Canada, China, India, France and Germany. Besides, it is used for biofuel and stock-feed production (Li et al., 2020). Due to the unprecedented growth of the world’s population, increased rapeseed yield is required to meet energy demands. The rapeseed generally bears pods (siliques) on the top of the raceme (primary stem) and side branches (Douglas and Riggs, 2005). After flowering, the rapeseed leaves senescence, falls off, and rely mainly on the green pods for photosynthesis. More than half of the dry matter for seed filling is provided by the pod valves/pericarp (Leng et al., 1992). Therefore, pod orientation plays a vital role in light energy absorption, which is required for photosynthesis and enhanced productivity. Based on the orientation of the pod on the raceme (pod angle), rapeseed can be classified into four types: straight, inclined, flat, and droop (Wu et al., 2007). The straight pod orientation is desired by rapeseed breeders, as they are not prone to ‘shading syndrome’. However, this pod architecture is rare in rapeseed germplasm (Sinhamahapatra et al., 2010). Pods of most cultivated Brassicas tend to be inclined, promoting the plant type to be more compact and disease build-up, especially to Sclerotinia stem rot, caused by the fungus *Sclerotinia sclerotiorum*.

Plant architecture significantly influences the photosynthetic efficiency and crop harvest index. The growth of plant organs in response to gravity is an essential factor that determines plant morphology. Most plant organs, especially those on mature plants, are displayed at a certain angle to the vertical growth axis and are not parallel to the gravity vector (Lorenzi and Perbal, 1990). Research has shown that the orientation of plant organs is controlled by the antagonistic interaction between the universal mechanism of gravitation and auxin-dependent transport (Roychoudhry et al., 2013).

Although the plant responses to gravity are yet to be fully understood, several key genes involved in the network of gravitational responses were identified in crop plants through genome-wide association studies (GWAS), map-based cloning and mutant studies. For example, in rice, *AGPL1*, *LPA1*, *ONAC106*, *PROG1*, *DWARF3*, and *TAC1* regulate tillering angle by transmitting gravity signals, suggesting polygenic inheritance (Morita et al., 2006; Okamura et al., 2013; Wu et al., 2013; Sakuraba et al., 2015 (Li et al., 2007; Tan et al., 2008; Sang et al., 2014). In addition, the middle creeping gene *LAZY1* also regulates the tillering and leaf angle by changing auxin distribution (Li et al., 2007). Genes such as *BRXL4*, *HSFA2D* and *OsPIN2* can regulate the expression of the *LAZY1* gene (Xu et al., 2005; Chen et al., 2012; Zhang et al., 2018; Li et al., 2019), and the mutation of the *SL* gene can inhibit the phenotype of *lazy1* by inhibiting the biosynthesis of auxin (Xu et al., 2005; Chen et al., 2012; Sang et al., 2014; Zhang et al., 2018; Li et al., 2019). In *Arabidopsis thaliana*, *LAZY1* and *TAC1* genes have been shown to regulate the branching angle, similar to rice (de Saint Germain et al., 2013; Waite and Dardick, 2018). In maize, *TAC1* can lead to an increase in branching angle (Ku et al., 2011). The genetic loci controlling the branch angle in rapeseed have been studied (Wang et al., 2016). However, the majority of studies focused on the branch angle of crops (Su et al., 2020; Wang et al., 2020; Yang et al., 2020), and few studies have addressed the role of the orientation of reproductive structures responsible for economic yields, such as pods, ears, and glumes (Li et al., 2012). In addition to branch angle, genes related to pod orientation also have been isolated and identified in the model plant *Arabidopsis thaliana*, tobacco, tomato and cucumber (Hobbie et al., 2000; Li et al., 2001; Venglat et al., 2002; Ha et al., 2007; Khan et al., 2012; Wang et al., 2015; Lin et al., 2016; Sun et al., 2019). The deletion mutation of KNAT1 gene produced downward pods (Venglat et al., 2002). The angle between the pod and raceme of auxin signal mutant axr6 was acute (Hobbie et al., 2000). Generally, the angle between wild-type pod and raceme is about 60°, and the overexpression of the *ROP2* gene of *Arabidopsis G* protein could reach the 90° or above (Li et al., 2001). In contrast, the inhibitory mutants were all smaller than 60° (Li et al., 2001). The pod angle of the actin filament bunching protein genes VILLIN2 and VILLIN3 double mutants vln2vln3 became smaller (van der Honing et al., 2012). The lateral organ boundary genes BOP1 and BOP2 proteins regulated the pod angle through *LBD* (Ha et al., 2007). The angle between pedicel/fruit and a raceme of spa mutant became smaller (Zhang and Yang, 2012). There are also many studies on the growth direction of Arabidopsis pedicels. Arabidopsis thaliana short pedicel mutant destroyed in KNAT1/*BP* gene showed decreased pedicel length and downward flowers (Douglas et al., 2002; Venglat et al., 2002). KNAT1/*BP* negatively regulates KNAT2, KNAT6 and ATH1 to ensure pedicels have a normal upward direction (Ragni et al., 2008; Li et al., 2012). It was found that NtSVP in tobacco, SlAGO7 in tomato and CsUp in cucumber control pedicel and fruit orientation (Wang et al., 2015; Lin et al., 2016; Sun et al., 2019).

GWAS combined with RNA-seq is an effective method to identify key genes which control plant traits (Bai et al., 2022). In this study, the GWAS was employed in a diverse set of rapeseed germplasm, and the associated regions for pod orientation were determined based on linkage disequilibrium (LD). Association between the favourable SNP alleles and pod orientation was assessed by the Mann-Whitney U test to identify SNP markers for marker-assisted selection in the rapeseed breeding programs. We further conducted RNA-seq analysis of pod pedicels of two rapeseed lines with significantly different pod orientations and prioritised candidate genes underlying QTL for pod orientation. Finally, bioinformatics analysis found the protein structure and sequence variation in the population for the candidate genes. Overall, this study aimed to provide a genetic basis of pod orientation in rapeseed so that ideotypes for plant architecture, optimal for increasing photosynthesis efficiency and yields, could be developed.

## 2 Materials and methods

### 2.1 Plant materials

One hundred and thirty-six rapeseed accessions were planted at three locations in Zunyi City, Guizhou Province (28°N, 107°E), Yangluo City, Hubei Province (30°N, 114°E) and Lu’an City, Anhui Province (32°N, 116°E) (**Supplementary Figure 1**). These three locations (environments) represented the upper, middle and lower regions of rapeseed cultivation in the Yangtze River valley in China. The experiment was conducted in randomised complete blocks with two replicates across each environment. Each accession was sown in a plot containing three rows with 54 individuals, spaced at 33 cm between rows and 11 cm between plants within each row, with a planting density of 270,000 plants/ha. All experiments were managed according to local field management and cultivation practices

### 2.2 Phenotyping

Two quantitative indices related to rapeseed pod orientation were measured: the angle of the pedicel on raceme (APR) and the angle of the pod on pedicel (APP) across 136 rapeseed accessions grown in three environments. We digitally measured the pod-related angles of a GWAS panel in three independent environments. We. At the maturity (BBCH scale 90), five plants of each rapeseed accession were selected from each environment investigation and measured for APR and APP using the image processing method described by Wang et al. (2015). A pod with raceme was cut from each plant and placed on a 20 × 20 cm blackboard for image analysis. A digital camera (DSLR-A350, Sony Inc, Japan), fixed on a tripod, was used to take a picture of the samples from the top, with a focal length of 35 mm. Digital images were downloaded output to the computer to draw the path and measure the angle with the AutoCAD package (https://www.autodesk.com.cn/).

### 2.3 Genotyping and population structure

A selected set of 136 rapeseed accessions were genotyped using the Brassica 60K Illumina Infinium**^@^** SNP array (Illumina) as described previously (Liu et al., 2016). After quality control (minor allele frequency>0.05 and missing data<20%) (Liu and Muse, 2005), a total of 21426 SNPs were selected for subsequent GWAS analysis. SNP density map was drawn using the CMplot package (Yin et al., 2021) in R3.3.3 (Team RC, 2013) to gauge the marker coverage across the *B. napus* genome. Principal component analysis was performed using Tassel v5.0 software (Bradbury et al., 2007). Only the first two principal components were plotted by the ggplot2 package (Wickham, 2011) in R3.3.3 (Team RC, 2013). MEGA-X software (Kumar et al., 2018) was used to draw the phylogenetic tree and show the genetic relationship among GWAS accessions. Both principal component analysis and phylogenetic tree are used to reveal the distribution of population structure. LD (linkage disequilibrium) attenuation was estimated to demonstrate the degree of genetic linkage, and it is drawn by PopLDdecay software (max decay distant = 2500, break = 6000bp, bin1 = 1000, bin2 = 6000) as described previously (Zhang et al., 2019).

### 2.4 Statistical and Genome-wide association analyses

The general linear model (GLM) was constructed using genotype, environment, and phenotype data. The ANOVA was carried out to determine whether the genotypic effect, environmental effect and genotype×environment interaction effect exist. The multi-environment mixed linear model (MLM) in Tassel5.0 software (Bradbury et al., 2007) was used to analyse the association between phenotype and genotype across three environments (Zunyi, Yangluo and Lu’an). The kinship relationship (K matrix) was added to the model to eliminate false positives caused by the genetic relationship. In this study, the *p*-value indicates whether an SNP was related to the corresponding trait, and *r^2^* shows the phenotypic variation explained by the marker. The standard of significant sites obtained by GWAS is *P*<0.0001. The Manhattan and QQ plots were drawn using the qqman package (Turner, 2014) in R3.3.3 (Team RC, 2013).

### 2.5 Gene functional annotation

All genes in the candidate region were annotated in the NR (https://ftp.ncbi.nlm.nih.gov/blast/db/FASTA/) and Swiss-port databases (https://www.expasy.org/resources/uniprotkb-swiss-prot) using blast software (Korf et al., 2003). The function of homologous genes in A. thaliana was screened and checked on the TAIR website (https://www.arabidopsis.org/index.jsp).

### 2.6 Evaluation of allelic effects

The QTL alleles that positively affected the smaller APR and APP were referred to as “favourable alleles”. In contrast, the alleles that cause the larger angles are referred to as “unfavourable alleles”. We selected the most significant SNP in each candidate region and selected favourable alleles for APR and APP. Because the phenotypes corresponding to marker genotypes did not conform to the normal distribution, we used a nonparametric test, the Mann-Whitney U test, to analyse the differences. Box plots were drawn by the ggplot2 software package (Wickham, 2011) in R3.3.3.

### 2.7 RNA-seq

**Figure 1:**
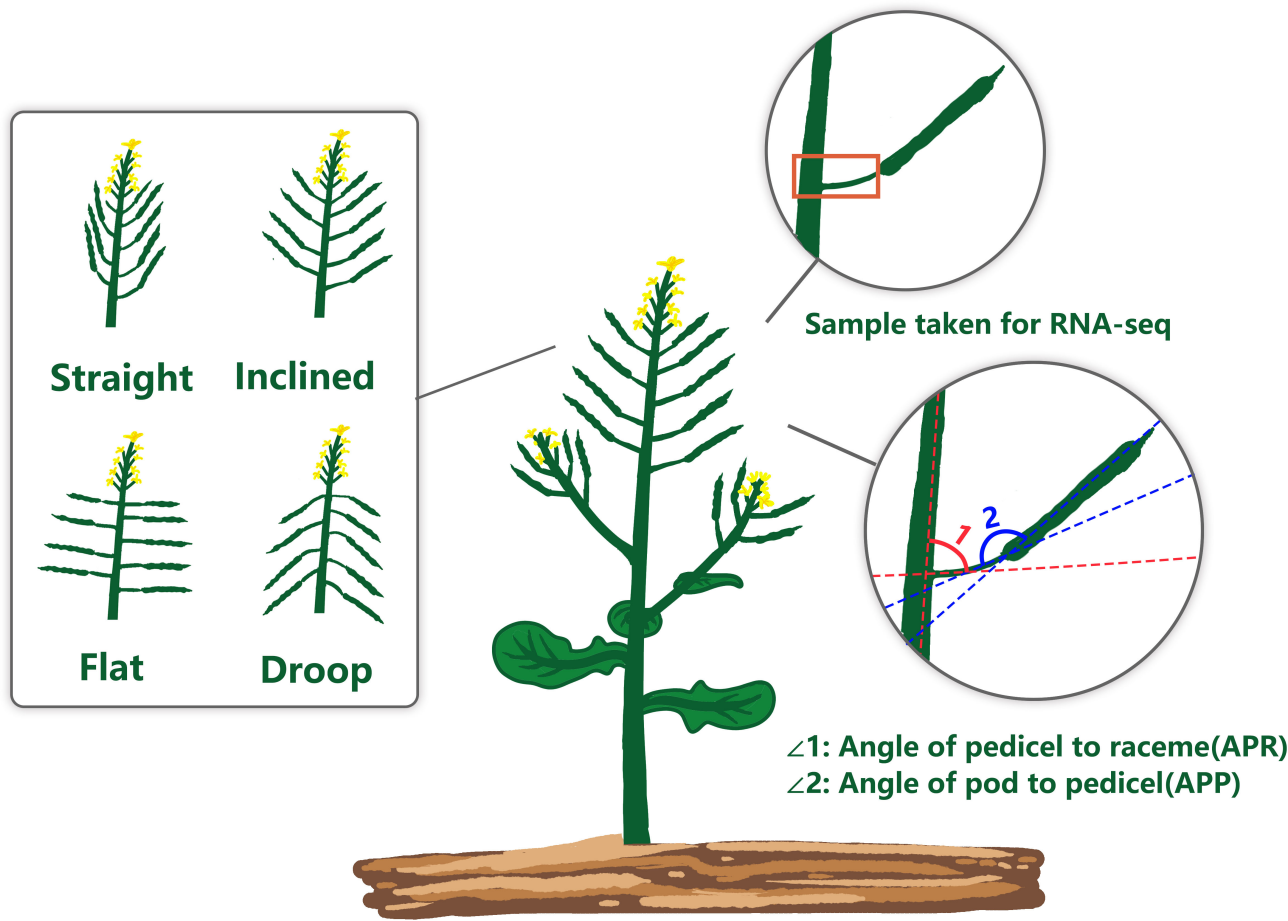
Schematic diagram of the rapeseed pod orientation showing two indices of pod orientation; APR, Angle of pedicel to raceme; and APP, Angle of the pod to the pedicel.

The pedicels of mature pods of rapeseed accessions: line 9319 (APR: inclined rapeseed, 59°, the APP is 170°) and line ZS11 (the APR of flat rapeseed, 90°, APP 170°) were collected for RNA sequencing (**Figure 1**). Both accessions were planted in triplicates under the controlled environment room maintained at 25°C light and dark regimes (16/8 h) till sample collection. RNA libraries were constructed using NEB Next^®^ Ultra™ RNA Library Prep Kit for Illumina (NEB, USA) following the manufacturer’s recommendations. Libraries were sequenced on Illumina HiSeq 4000 platform, resulting in 150 bp paired-end reads. The quality control of RNA-seq reads was carried out by fastqc (Andrews, 2010) software and summarised by MultiQC (Ewels et al., 2016). For RNA degradation, the transcript integrity index (TIN) was checked using RSeQC following the method described by Wang et al. (2012). After quality control and filtration, the transcriptome sequencing Reads were compared to the reference genome assembly of ZS11 (version 20200127) using hierarchical indexing for spliced alignment transcripts (HISAT) version 2.1.0 (Kim et al., 2015). StringTie software v1.3.6 (Pertea et al., 2015) was used to assemble new transcripts. RSEM v1.2.31 was used to quantify the gene expression level. The accuracy and sensitivity of mutation detection using GATK Best Practices (O'Connor and Van Auwera, 2020) were similar to those using SAMtools mpileup (Li et al., 2009). Considering the rapidity and convenience of mutation detection by SAMtools mpileup, sample mutation detection was adopted by SAMtools mpileup based on the comparison data between HISAT2 and genome, and the results were stored in VCF format.

### 2.8 Identification of gene families

We downloaded candidate protein sequences involved in the pod orientation of different species from the NCBI website (https://www.ncbi.nlm.nih.gov/) and established a local protein database. Seven genes of IGT families in *Arabidopsis thaliana* were compared with the local protein database by BLAST (Ye et al., 2006). Mafft software (Katoh et al., 2002) was used to align the IGT family genes, and finally, the evolutionary tree was constructed using FastTree software (Price et al., 2009). SAUR gene family had hidden Markov model (PF02519) on the Pfam website (http://pfam.xfam.org/), so we used Hmmer (Finn et al., 2011) software to identify the SAUR gene family, and also used Mafft software to align the SAUR gene family, and then used FastTree software to construct the phylogenetic tree. The NWK format file obtained from tree building is used in R to draw the unrooted phylogenetic tree by TreeAndLeaf (Cardoso et al., 2022) package. Different species were depicted as different colours of evolutionary leaves, and the genetic relationship with rapeseed was used as the size of evolutionary leaves. The distance of genetic relationship was referred to the Taxonomy Common Tree of NCBI (https://www.ncbi.nlm.nih.gov/).

### 2.9 Protein structure prediction

Alphafold2 (Jumper et al., 2021) software can predict the protein structure with 90% accuracy. The protein structure was predicted by alphafold2 software, and the rank_0 model with the best prediction score was selected from the rank_0 to 4 models of the predicted results to draw the protein structure. ChimeraX software (Pettersen et al., 2021) was used to draw protein structure, calculate B-factor value and map to protein structure.

### 2.10 Nucleotide diversity

The *Pi* value of SNP diversity was calculated from 289 rapeseed core accessions collected worldwide (Xuan et al., 2020). The original data of the Next-generation Sequencing was downloaded from the NCBI website (https://www.ncbi.nlm.nih.gov/SRA/SRP15312). The sequences were compared to the reference genome of ZS11 (Chen et al., 2021) by Sentieon software (Freed et al., 2017), and the GVCF file was used for detection variation among 289 accessions. GATK software was used to identify GVCF variant files as SNP and InDel VCF files (O'Connor and Van Auwera, 2020). According to the distribution of each parameter in the population, GATK software was used to control the data quality of the variant file, and the filter parameter used by the SNP file is QD < 2.0; FS > 50; MQ < 20; MQ Rank Sum > -12.5; Read Pos Rank Sum > -8.0. Vcftools software (Danecek et al., 2011) was used to calculate diversity. A 100-bp sliding window with a 25-bp step size was used to calculate nucleotide diversity. Using R language, the nucleotide diversity of the gene and its upstream and downstream 2Kb regions was fitted by the loess method, with a span of 0.3.

## 3 Results

### 3.1 Phenotypic variations for APR and APP

**Figure 2:**
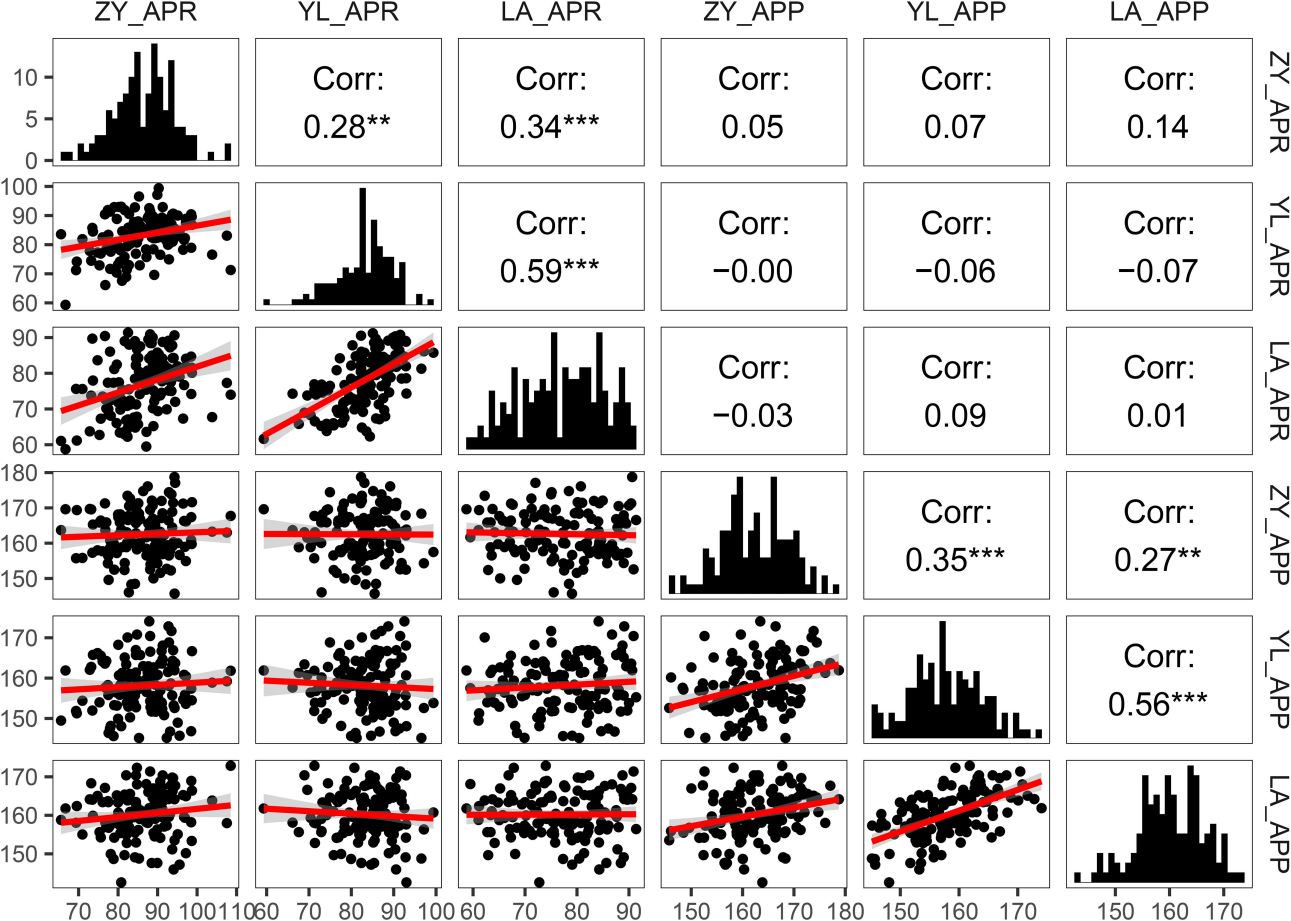
Pearson correlations and frequency distribution for pod orientation traits (Angle of the pedicle to raceme: APR and angle of the pod to pedicel: APP) among 136 rapeseed accession grown across three environments (ZY: Zunyi, YL: Yangluo, LA: Lu’an). The lower left part of the picture is the scatter diagram among the environments; the red line shows simple linear regression of variables. The diagonal corner is the frequency distribution diagram of the environments. The upper right corner is the correlation between the environments. **: P<0.01 indicates highly significant correlation; ***: P<0.001 indicates extremely significant correlation.

To determine the extent of natural variation in pod orientation, as APR and APP (**Figure 1**), 136 rapeseed accessions were grown in the field across three different environments. Statistical analysis showed that the variation in APR was due to genotypic (G) and environmental effects (E), but no G × E interaction existed. However, the effects of G, E and G × E were significant for the APP (**Table 1**). Broad sense heritability (*H^2^*) was calculated, and it ranged from 49.65% (APR) to 76.65% (APP). The frequency distribution of the APP and APR showed continuous variation (**Figure 2**), suggesting that the pod angle-related characteristics were inherited quantitatively.

**Table 1:** Summary statistics on the phenotypic variation of pod orientation measured as Angle of the Pedicle to Raceme (APR) and Angle of the Pod to Pedicel (APP) in the 136 rapeseed accessions grown across three environments: ZY: Zunyi; YL: Yangluo, and LA: Lu’an.

Measure for pod orientation	Environment	Angle range (°)	Mean (°)	Standard Deviation	Variance	G	E	G×E	*H* ^2^ (%)
APR	ZY	66.00-109.00	86.20	7.80	60.85	0.000**	0.044*	0.894	49.65
	YL	59.32-99.38	83.20	6.72	45.18				
	LA	59.00-91.00	76.94	8.27	68.34				
APP	ZY	145.70-178.75	162.56	6.77	45.81	0.001**	0.000**	0.021*	75.65
	YL	145.16-174.06	158.16	6.24	38.97				
	LA	142.59-172.86	160.22	6.04	36.46				

Significance for the effect of genotype (G), environment (E), and genotype by environment interaction (G × E) on phenotypic variance estimated by ANOVA across three environments (*: P<0.05 indicates significant; **: P<0.01 indicates highly significant).

Genetic variation for pod orientation among 136 accessions of rapeseed is presented (**Figure S3**). The APR varied from 66° to 109°, 59.32° to 99.38°, and 59° to 91° with the mean values of 86.20 ± 7.80°, 83.20 ± 6.72° and 76.94 ± 8.27° in the ZY, YL and LA environments, respectively. Most accessions (80%) had APR ranging from 73° to 89°. The Australian cultivar, Rivette and F459, had a small APR angle (APR<70°), while Cibrabra and OG3186 had a large APR angle (APR>90°). The Australian variety, Rivette, had the minimum APR (59.32°), while the European variety, Nilla, had the maximum APR (109°). The APP varied from 142.59° to 178.75° across environments (**Table 1**). The 80% germplasm accessions had APP varying from 153° to 167°. Rapeseed accessions: BLN3342 and R11 had small APP angles (APP<150°), whereas OG3190 and P10 had a large APP angle (APP>170°).

We ranked rapeseed accession based on their angles to identify accessions of interest for rapeseed breeding. In this study, we defined the pod orientation as the sum of APR and APP less than 200° as straight type; greater than 200° and less than 250° were inclined types; more than 250° and less than 300° degrees were flat type, and greater than 300° was droop type (**Figure 2**). We classified pod orientation based on their angle into discrete categories: straight type with the APR of ~ 0° and the APP of ~ 180°; the inclined type having an APR of ~ 45° and the APP of ~ 180°; the flat type with APR of ~ 90°, and the APP of ~ 180°; droop type which had APR and APP of ~ 180° and 180°, respectively, Most accessions had inclined-type pods while none had straight and drooped types pods (**Supplementary Figure 4**).

Pearson correlation analysis between angle-related traits (APR and APP) across different environments showed significant positive correlations (*r* = 0.27 to 0.59, **Figure 2**). These results suggested that at least part of the genetic control of variation in angle-related traits was due to the environments (Zunyi, Yangluo and Lu’an). There was no relationship between APR and APP across three environments (*r* = -0.06 to 0.05), which indicated that different genetic factors might control variation for both measures of pod orientation.

### 3.2 Genome-wide association analysis

**Figure 3:**
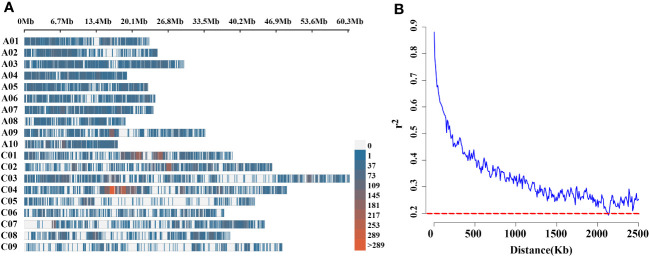
**(A)** High-density physical map of 136 rapeseed accessions, revealed with *Brassica* 60K Illumina Infinium^@^ SNP array. Different colours indicate the SNPs contained in the 1Mb genomic region. The red indicates SNPs rich region, the blue area indicates low-density SNP regions and the gray area indicates the absence of SNP markers; **(B)** LD decay of *Brassica* genome of 136 natural population using 60K Illumina Infinium^@^ SNP array. The dotted line in red colour shows that r^2^ is 0.2. X-axis: physical distance is shown in Kb.

To uncover the genetic basis of pod orientation, the significance of the associations between phenotypes and 21,426 genome-wide SNPs markers (Liu et al., 2016) was evaluated using the linear mixed (Q+K) model. There were 8,878 polymorphic SNPs (41.44%) in the A sub-genome and 12,548 (58.56%) in the C sub-genome, covering 238.13 Mb and 405.53 Mb sub-genomes, respectively, among the 136 rapeseed accessions (**Supplementary Table 1**). SNPs were evenly distributed across the A sub-genome and C sub-genomes (**Figure 3A**). The average LD decay of sub-genome A was smaller than that of sub-genome C. Under the threshold of *R* ^2^ = 0.5, the LD decay of the whole population was about 147 kb, and under the threshold of *R* ^2^ = 0.2, it was about 2.1 Mb (**Supplementary Table 2**, **Figure 3B**).

**Figure 4:**
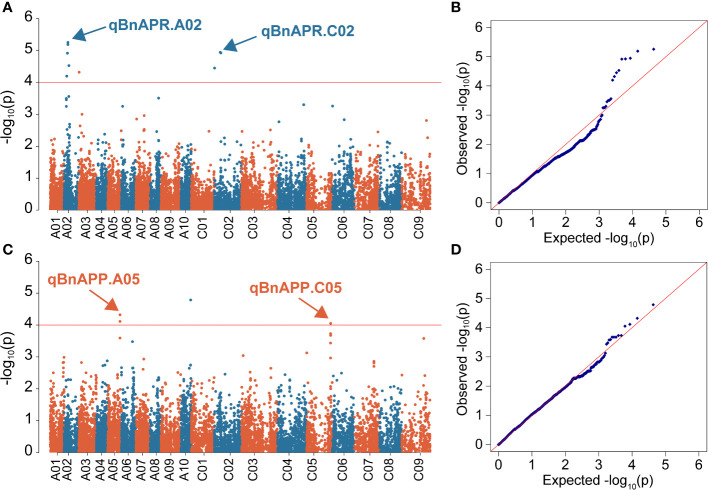
Manhattan and quantile-quantile (QQ) plots showing associations between SNPs and two pod orientation traits in 136 rapeseed accessions. GWAS was conducted by the mixed linear model (MLM) in Tassel v5.0. **(A)** Manhattan plot for the angle of pedicel to raceme (APR) across three environments; **(B)** Q-Q plot for the APR across three environments; **(C)** Manhattan plot for the angle of the pod to pedicel (APP); **(D)** Q-Q plot for APP. The horizontal red line represents the genome-wide significant threshold (*p*=1.0 × 10^-4^) For clarity, only SNPs with a −log_10_P-value > = 4 are shown. SNPs are plotted according to their physical positions on the ZS11 reference genome.

Manhattan and Quantile-Quantile (Q-Q) plots showed significant genetic associations for APP and APR across environments (**Figure 4**). Using the -log10(P) ≥4 as threshold LOD, we identified four QTL, designated as qBnAPR.A02, qBnAPP.A05, qBnAPR.C02, and qBnAPP.C05 on chromosomes A02, A05, C02, and C05, respectively (**Table 2**, **Table S3**). Interestingly, qBnAPR.A02 and qBnAPR.C02 were located in the homologous regions on the group 2 chromosomes (A02/C02), while qBnAPR.A05 and qBnAPP.C05 were mapped to homologous regions on A05/C05 chromosomes (**Supplementary Figure 5**). SNP markers accounted for 4.50% (APR) to 16.78% (APP) of the phenotypic variance (**Table 2**). In addition to four significant QTL, two minor QTL (one for APR on the A08 and one APP on chromosome A06) were also detected (original data not shown). However, these QTL had low LOD scores (LOD>3, but <threshold LOD 4). Two false QTL (one for APR on the A03 chromosome and one APP on chromosome A10) were excluded because only one SNP was significant at these loci.

**Table 2:** QTL for pod orientation (Angle of the Pedicle to Raceme: APR and Angle of the Pod to Pedicel: APP) identified in a panel of 136 accessions of *B*.

Phenotypic measure	QTL	Peak SNP	Chromosome	Physical Position on ZS11 genome	-log_10_(P) value	*R* ^2^ (%)	Genome Region in LD (bp)
APR	qBnAPR.A02	Bn-A02-p9706613	A02	9231713	5.26	4.5	8807713-9655713
	qBnAPR.C02	Bn-scaff_17725_1-p505212	C02	10138679	4.95	4.3	7638679-12638679
APP	qBnAPP.A05	Bn-scaff_17441_3-p165957	A05	41469899	4.32	16.78	41045899-41893899
	qBnAPP.C05	Bn-scaff_17441_3-p68051	C05	54171343	4.05	15.66	51624343-56618343

QTL were marked with SNP markers mapped onto the ZS11 reference genome assembly. QTL were identified at -log(_10_) ≥ 4, and for identification of genome region. The average LD decay of sub-genome A was smaller than that of sub-genome C. Under the threshold of R^2^ = 0.5 the LD decay of the whole population was about 147 kb and under the threshold of R^2^ = 0.2, was about 2.1Mb).Angle of pedicle to raceme: APR and Angle of pod to pedicel: APP. *napus* using a genome-wide association approach.

**Figure 5:**
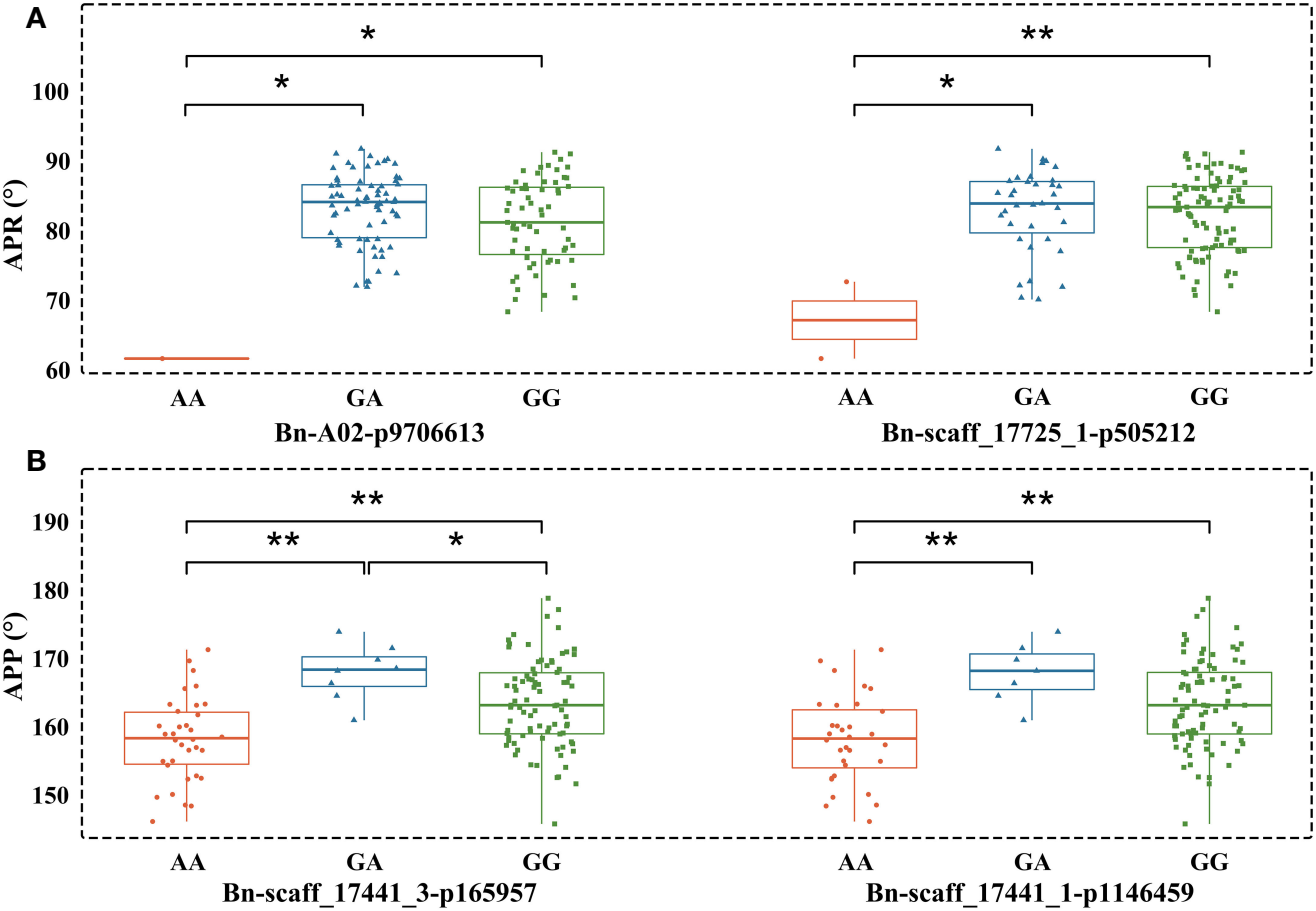
Boxplots showing the association between alleles of two peak SNP markers and pod orientation. **(A)** Angle of the pod on raceme (APR) and corresponding SNP alleles; **(B)** Angle of the pod on pedicel (APP) and corresponding alleles. All statistical associations are marked with *(at *P*<0.05) and with ** (at *P*<0.01).

We observed that phenotypes corresponding to different genotypes did not show normal distribution. Therefore, the Mann-Whitney U test (**Supplementary Table 4**) was applied to determine whether the phenotypes (different measures of pod orientation) correspond to marker alleles. The AA SNP allele of Bn-A02-p9706613 decreased the APR compared to GA and GG. Similar results were observed with the AA allele of Bn-scaff_17725_1-p505212, which showed significant differentiation from large pod angle accessions with GA or GG alleles. AA alleles at Bn-scaff_17441_3-p165957 and Bn-scaff_17441_1-p1146459 also showed an association with reduced APP compared to GA and GG alleles (**Figure 5**).

### 3.3 Candidate genes associated with pod orientation

**Figure 6:**
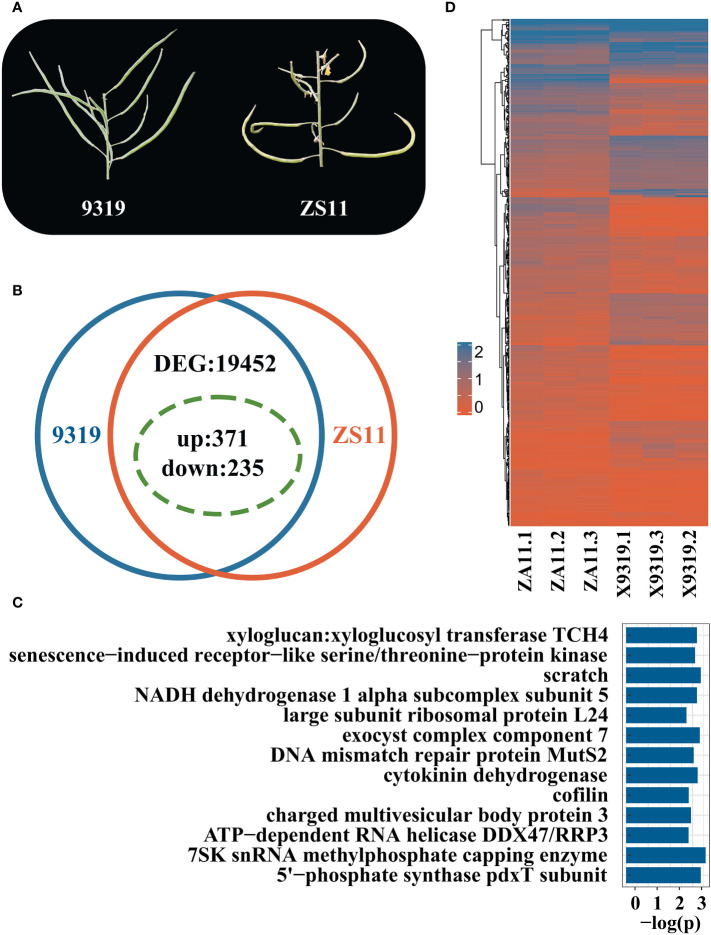
RNA-seq analysis of rapeseed lines with contrasting pod orientation phenotypes. **(A)** Phenotypic difference of pod orientation between 9319 line (Angle of the pedicle to raceme (inclined type, 59°, the angle of the pod to pedicel is 170°) and ZS11 line (Angle of pedicel to raceme, flat type, 90°; Angle of the pod to pedicel:170°); **(B)** Statistics summary of differentially expressed genes (DEGs) between 9319 and ZS11. The green dotted circle indicates the DEGs in QTL; **(C)** Enriched KEGG pathways of candidate DEG between 9319 and ZS11; **(D)** Heat map of candidate genes of DEG expression, the expression levels were transformed by log_10_(x+1), 0 represents that the gene was not expressed, and 2 represents that the expression level was high.

We selected two accessions that had extreme phenotypes: line 9319 (APR: inclined rapeseed, 59°, the APP is 170°) and line ZS11 (the flat rapeseed, APR of 90°, APP 170°) (**Figure 6A**). The DEGs were analysed in both lines with extreme phenotypes by RNA-seq approach (**Supplementary Table 5**). Through RNA seq analysis, 128 million reads were generated. The data of each library ranged from 5.8 Gb to 8.2 Gb, and the Q30 base calling was ~95%. 85% of the reads were mapped to the reference ZS11 Genome.

A total of 19,452 DEGs were identified between 9319 and ZS11 accessions, including 12,205 up-regulated genes and 7,247 down-regulated genes (**Figures 6B, C**). Of them, 606 DEGs were identified, underlying the QTL regions for pod orientation. Among 606 candidate genes, 235 DEGs were down-regulated, and 371 were up-regulated (**Figure 6B**). We detected 292 DEGs underlying qBnAPR.A02 and 314 DEGs underlying the qBnAPR.C02 (**Supplementary Table 6**).

KEGG enrichment analysis of 66 genes showed that the different candidate genes involved 309 pathways (**Supplementary Table 7**) and enriched for 13 pathways, including K00279 (**Figure 6D**). Among them, cytokinin dehydrogenase and xylloglucan: xylloglucosyl transferase TCH4 pathways are related to plant growth and development. Cytokinin dehydrogenase can promote the differentiation and growth of buds (Zhang et al., 2008). Xyloglucan: xyloglucosyl transferase TH4 plays an important role in the formation and reconstruction of cross-linking in xyloglucan and participates in the plant cell wall modification process (Jan et al., 2004). Annotations revealed 7 genes among 606 candidate genes related to auxin: *BnaC02G0079400ZS*, *BnaC02G0175200ZS*, *BnaC02G0118700ZS*, *BnaC02G0102400ZS*, *BnaC02G0050100ZS*, *BnaC02G0166500ZS*, and *BnaA02G0138300ZS*.

We focused on three interesting DEGs which underlie QTL for pod orientation: the first two are homologous genes related to auxin synthesis. BnaA02G0138300ZS and BnaC02G0175200ZS, designated as *BnSAUR32.A02* and *BnSAUR32.C02*, respectively. The third gene was BnaA02G0200300ZS, which was related to root angle growth in *Arabidopsis thaliana* (Yang et al., 2020). *BnaA02G0200300ZS* was designated as *BnLAZY4.A02. BnSAUR32.A02* and *BnSAUR32.C02* genes are homologues that map in the proximity 1604 kb and 654 kb upstream of the peak SNP of the APR trait (**Table 3**). The *BnSAUR32.A02* is a homologue of *SAUR32* (*AT5G53590*) encoding SAUR-like auxin-responsive protein in *Arabidopsis*. *BnSAUR32.C02* and *AT5G53590* had poor homology and are *SAUR32-like* genes.

**Table 3:** Candidate genes for pod orientations prioritised by combining GWAS and RNA-seq results.

Gene	ZS11 ID	9319-fpkm	ZS11-fpkm	FDR	Physical position from the peak SNP (Kb)
*BnSAUR32.A02*	*BnaA02G0138300ZS*	23.70	10.20	8.73E-03	- 1604
*BnSAUR32.C02*	*BnaC02G0175200ZS*	73.58	18.13	6.19E-09	- 654
*BnLAZY4.A02*	*BnaA02G0200300ZS*	1.22	0.00	4.80E-05	+ 3332

FPKM and FDR refer to fragments per kilobase of exon model per million mapped fragments and false discovery rate, respectively.

### 3.4 Phylogenetic and nucleotide diversity analyses of candidate genes

**Figure 7:**
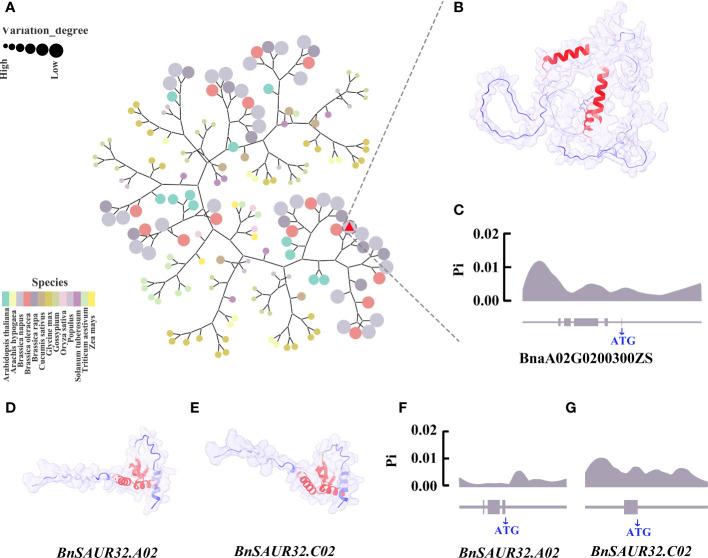
Bioinformatics analysis of three key candidate genes **(A)** Phylogenetic tree of IGT gene family in 13 species, including *Arabidopsis thaliana, Arachis hypogaea, Brassica oleracea, Brassica napus, Brassica rapa, Cucumis sativus, Glycine max, Gossypium, Oryza sativa, Populus, Solanum tuberosum, Triticum aestivum and Zea mays*. Different colours represent different species, the size of the circle represents the genetic relationship with rapeseed, and the larger the circle, the closer it is to rapeseed. Red triangle marked *LAZY4*. **(B)** Prediction of *BnLAZY4.A02* protein structure using Alphafold2. The blue the protein colour, the lower the B-factor, and the red the protein colour, the higher the B-factor. **(C)** The nucleotide diversity distribution of *BnLAZY4.A02* in 289 core germplasm collections; **(D)** Protein structure map predicted by *BnSAUR32.A02* gene. The bluer the protein colour, the lower the B-factor, and the red the protein colour, the higher the B-factor; **(E)** Protein structure map predicted by *BnSAUR32.C02* gene; **(F)** Distribution of nucleotide diversity of *BnSAUR32.A02* gene in 289 core germplasm collections; **(G)** Distribution of nucleotide diversity of *BnSAUR32.C02* gene in 289 core germplasm collections.

The *BnLAZY4.A02* gene belongs to the IGT gene family, with 167 IGT family genes in 13 species. To investigate the relationship of *BnLAZY4.A02* that was expressed in the pedicel of line 9319 but not in the pedicel of line ZS11, with other species, we carried out cluster analysis using neighbour-joining approach (**Figure 7A**). *BnLAZY4.A02* was clustered on the same branch as Chinese cabbage and cabbage, which is consistent with the genetic lineage of *Brassica* species. There were three helical structures in the *BnLAZY4.A02* protein with high B-factor values, which may regulate upstream and downstream genes (**Figure 7B**).

To gain insights into nucleotide diversity in candidate genes involved in pod orientation, we utilised public genomic sequence data of 289 rapeseed core collections (Xuan et al., 2020). Results showed that the nucleotide diversity peak appeared downstream of the *BnLAZY4.A02* gene (**Figure 7C**), which could regulate the expression of this gene and hence, responsible for genetic variation for pod orientation in rapeseed. Further research is required to substantiate this proposed hypothesis. *BnSAUR32.A02* and *BnSAUR32.C02* genes belong to the SAUR gene family. We identified 1,857 SAUR family genes by gene family analysis in 13 species, including *Arabidopsis thaliana*, *Arachis hypogaea*, *Brassica oleracea*, *Brassica napus*, *Brassica rapa*, *Cucumis sativus*, *Glycine max*, *Gossypium*, *Oryza sativa*, *Popular*, *Solanum tuberosum*, *Triticum aestivum* and *Zea mays* (**Supplementary Figure 6**). The protein structures of *BnSAUR32.A02* and *BnSAUR32.C02* genes were very similar (**Figures 7D, E**). The active region of the protein was concentrated in the centre of the protein. In the core collection of 289 rapeseed accessions, the nucleotide of *BnSAUR32.A02* and *BnSAUR32.C02* genes had almost no variation, and its Pi value was low, 0.0001 (The critical value of nucleotide diversity is 0.005.) (**Figures 7F, G**). However, there was considerable nucleotide diversity *(Pi* value: 0.0087, Pi>0.005), in the upstream region of *BnSAUR32.A02*, which could affect the gene expression.

## 4 Discussion

Crop architecture is an important physiological and agronomic trait that could drive rapeseed yield. The branch angle determines the overall plant shape, while the pod angle contributes to the photosynthesis rate. The ideal plant architecture can make the best use of resources such as light, water and nutrients, achieving the maximum yield and possibly the best quality. In recent years, crop architecture has become a vital breeding aim of genetic improvement programs. Investigating pod orientation *via* GWAS expands the research in two ways. First, this study mined the natural variation in pod orientation traits in a diverse GWAS panel of accessions rather than two parents, used in most biparental QTL mapping studies. Secondly, we identified four QTL and candidate genes associated with pod orientation.

### 4.1 Genetic variation for pod orientation in rapeseed

Earlier studies investigated genetic variation for pod orientation using qualitative scores: straight, inclined, flat and droop (Wu et al., 2007). However, these assessments are subjective and less accurate for genomic analysis. In this study, we followed a more accurate technique, employing image processing to assess genetic variation in pod orientation (APP and APR). This approach avoided errors in the manual angle measurement under field conditions. Moreover, we used quantitative scores to classify pod orientation. We only found accessions with flat and inclined types in our GWAS panel. Further research is required to screen national and international diverse accessions for straight types desired by the rapeseed improvement programs. Moderate correlations across some environments suggest that pod orientation traits can be selected in rapeseed breeding and are suitable for selection in the early generation.

### 4.2 Four QTL for pod orientation

There were statistically significant G×E interactions for both APP. APR across environments. Therefore, we employed the multi-environment-based MLM model, thus excluding the impact of the environment on the genotype-phenotype association, as opposed to the approach followed by (Raman et al., 2022). We identified four significant QTL regions that accounted for a small proportion of variation (4.5 to 17%). These results suggest that variation in pod orientation is polygenic and influenced by the environment. In this study, we did not identify QTL associated with QTL × Environment interaction.

To identify favourable alleles for pod architecture, we sought an association between SNP marker alleles (**Figure 5**) and phenotypes. The flat type of pods occupies considerable space, is more prone to shading, limiting the photosynthetic area and is not suitable for high-density cultivation. While the straight or inclined type pods can maximise the function of light energy and space utilisation and have more advantages. Based on the above assumption, we defined the alleles that promote pod orientation to straight or inclined as the favourable allele. The AA marker genotype is a favourable allele for short pod angle on peak SNPs of four significant loci. qAPR.A02 delimited with Bn-A02-p9706613 marker and qAPP.A05 delimited with Bn-scaff_17441_3-p165957 marker cumulatively contributed 21.28% variation in pod orientation. GG alleles detected with SNPs markers (Bn-A02-p9706613 and Bn-scaff_17725_1-p505212) are in the smallest inclined type materials. Two SNPs, Bn-scaff_17441_3-p165957 and Bn-scaff_17441_3-p68051, are AA genotypes, and not all four loci are favourable alleles of pod orientation. On this basis, the germplasms of inclined type with GG alleles of qBnAPP.A05 and qBnAPP.C05 might be improved to AA alleles obtain straight type.

### 4.3 Candidate genes for rapeseed pod orientation

We followed two approaches to identify the candidate gene underlying QTL for pod orientation. Firstly, we examined genomic regions in LD (upstream and downstream 424 Kb for the A subgenome and 2.5Mb for the C subgenome). Secondly, we identified DEG underlying QTL regions. We also searched *Arabidopsis thaliana priori* candidate genes whose role in branch and inflorescence angle has been confirmed.

We prioritised three candidate genes: *BnLAZY4.A02*, *BnSAUR32.A02* and *BnSAUR32.C02*, underlying pod orientation QTL (**Table 2**). *BnLAZY4.A02* was expressed in the pod pedicel of 9319 but not in the pod pedicel of ZS11. The *BnLAZY4.A02* gene belongs to the *IGT* family, which plays a role in the gravity signals of several plant species (Waite and Dardick, 2021). *LAZY* homologues have been shown to regulate shoot branching angles in *Arabidopsis*, rice, maize and several perennial tree crops (Roychoudhry and Kepinski, 2015). In *Arabidopsis thaliana*, recent studies have shown that increasing LAZY4 expression in pifq mutants can partially save the interrupted hypocotyl gravity phenotype of dark growing IFQ but cannot save the development of endoderm amyloplast (Yang et al., 2020). During the growth in *Arabidopsis*, amyloplast in columella cell sank due to gravity, and LAZY protein regulated auxin transport in a polarised way after feeling this change. The polar transport of auxin leads to anti-gravity growth (Furutani et al., 2020). Therefore, the mechanism of LAZY4 regulating pod angle may be the same. In a recent study on the effect of jasmonic acid on the lateral root orientation of *Arabidopsis thaliana*, the transcription activation of *LAZY4* mediated by a jasmonic acid-related gene *MYC2* also indicates that the *LAZY4* gene is related to plant growth angle (Sharma et al., 2022).

Genetic variation in pod orientation QTL on A02 and C02 (**Table 2**) could be conditioned by small auxin up-regulated RNA genes (*SAURs*) homologues: *BnSAUR32.A02* and *BnSAUR32.C02*. S*AURs* are implicated in regulating dynamic and adaptive growth in *Arabidopsis* and sunflower (Atamian et al., 2016; Bemer et al., 2017; Stortenbeker and Bemer, 2019). Auxin and other upstream factors can also regulate the expression of the *SAURs* gene (Favero et al., 2017; van Mourik et al., 2017; Hu et al., 2018). *SAURs* can interact with PP2C.D phosphatases (Spartz et al., 2014) to induce plant growth by regulating cell wall acidification. *SAURs* can also act independently of auxin. Zhou et al. identified 98 *SAUR* gene families in apple, and screened 25 QTLs for regulating root growth angle. Seven *SAURs* were selected as candidate genes for regulating root growth angle through protein-protein interaction network (Zhou et al., 2022). In the leaf angle of sorghum, a strong candidate gene SAUR36 was identified by RNA-seq, and its expression may increase the leaf angle (Natukunda et al., 2022). It is worth noting that the variation of the promoter region of *BnSAUR32.A02* could also regulate genetic variation in pod orientation underlying QTL on A02 and C02 chromosomes.

In conclusion, we determined genetic variation in pod orientation in diverse rapeseed accessions. Utilising GWAS, we identified four significant QTL for pod orientation on A02, A05, C02, and C05 chromosomes in diverse rapeseed accessions. In addition, we performed RNA-seq analysis and prioritised three candidate genes: differentially expressed between inclined and flat pods and localised within the QTL associated with APP and APR. Thus, our GWAS and transcriptome studies delineated three putative candidate genes underlying pod orientation in *B. napus*. Overall, the results of this study provided the genetic tools and resources for understanding the molecular basis of variation in pod orientation of rapeseed. These findings may facilitate the development of and cultivation of rapeseed ideotypes.

## Data availability statement

The datasets presented in this study can be found in online repositories. The names of the repository/repositories and accession number(s) can be found below: https://ngdc.cncb.ac.cn/?lang=en, CRA007893.

## Author contributions

JL designed and supervised the study. WW and JL collected the samples. QH provided the germplasm. YY performed genetic, and transcriptome data analyses. YY, HR and JL discussed results and prepared the manuscript. All authors contributed to the article and approved the submitted version.
