# Supplementary material for: Genome-wide association and RNA-seq analyses identify loci for pod orientation in rapeseed (Brassica napus)
Source: Front Plant Sci. 2023 Jan 13;13:1097534. doi: 10.3389/fpls.2022.1097534 (PMC9880488; doi:10.3389/fpls.2022.1097534)
Supplement: Supplementary file 1 [file DataSheet_1.docx]

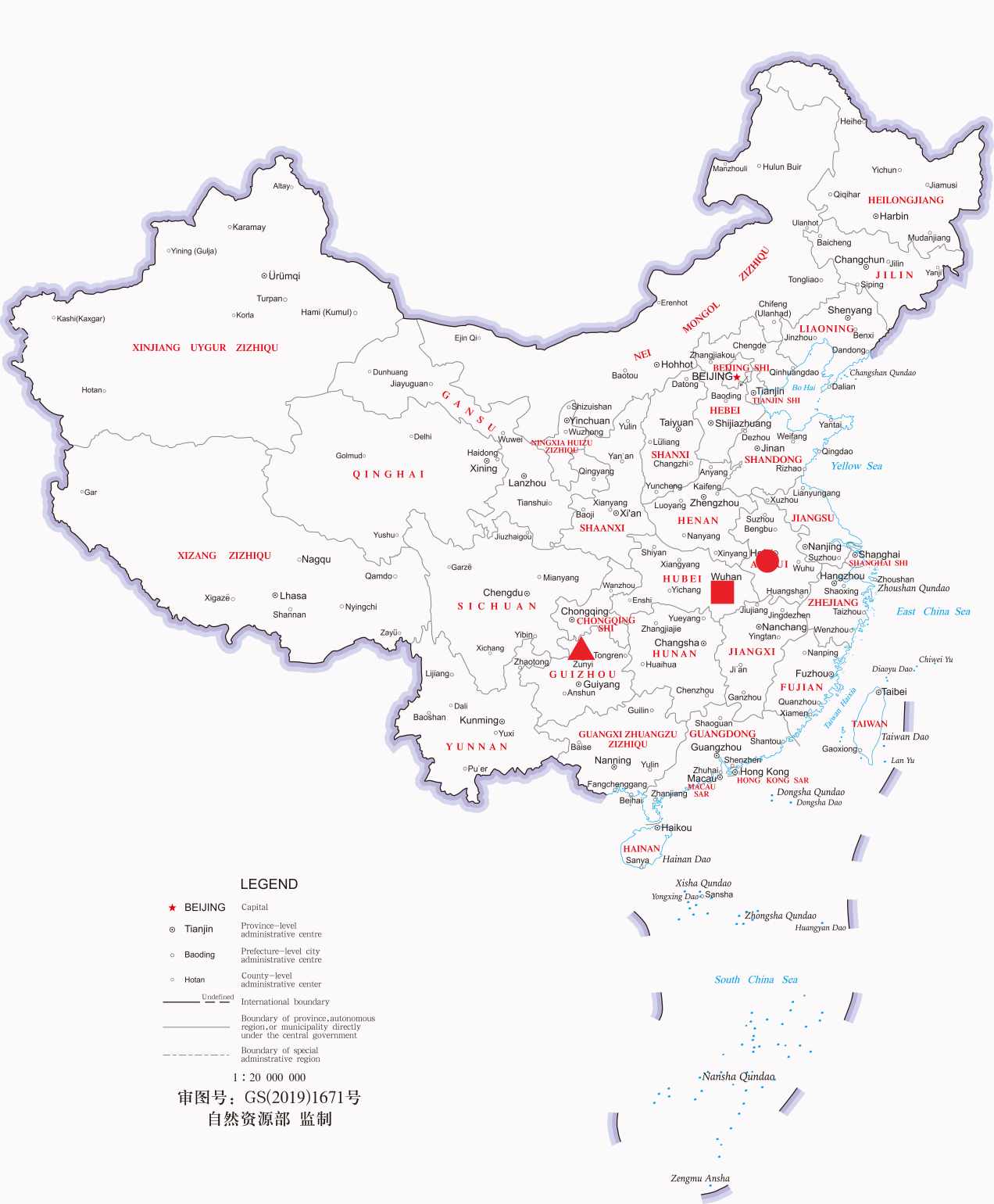


Fig. S1: The loction of three environment (Zunyi city: red triangle; Yangluo city: red square and Lu'an city: red circle) were shown in the Chinese national map.


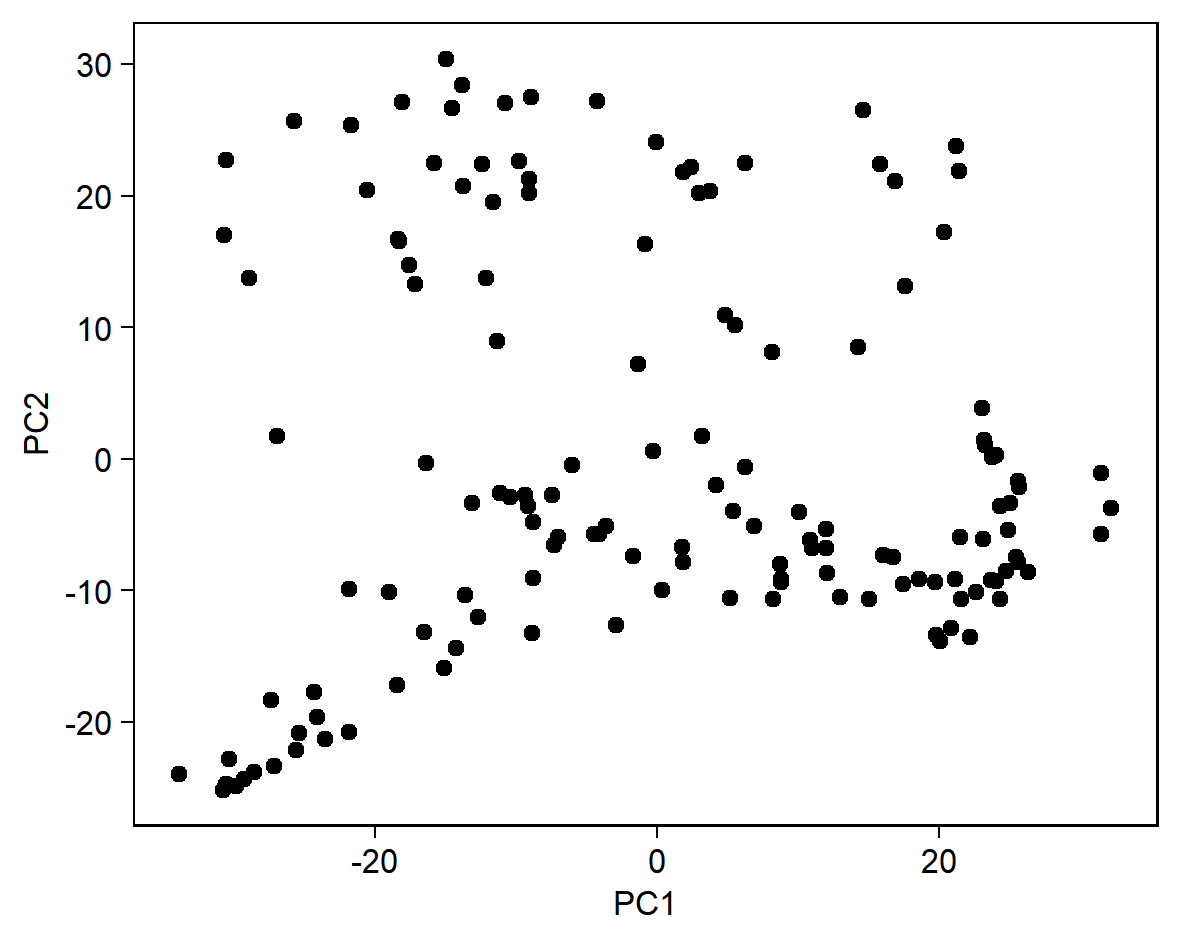


Fig. S2 Principal components analysis (PCA) of the GWAS (genome-wide association studies) panel of 136 accessions of *B. napus,* Principal component 1 (PC1) explained 9.24% variation, PC2 explained 6.30% of the variation.


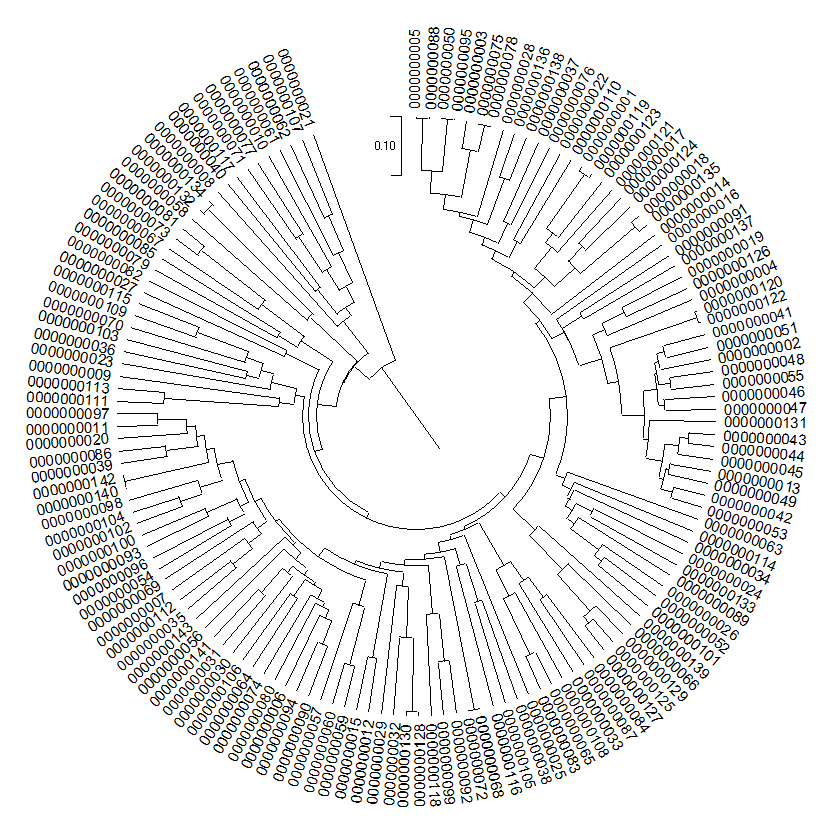


Fig. S3: Neighbor-joining (NJ) tree showing grouping of *Brassica napus* accessions used forGWAS (genome-wide association studies). Details of accession are given in Supplementary Table 8.


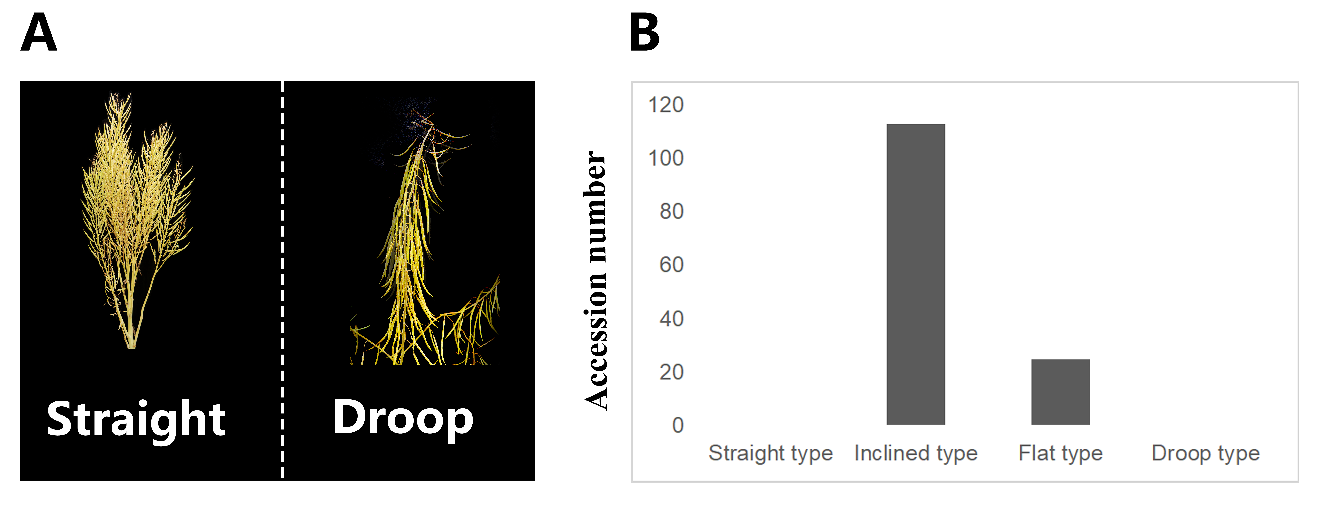


Fig. S4: Genetic variation for pod orientation type in *Brassica napus.* A: Morphological features of straight and drooped type of rapeseed. B: Histogram of pod orientation of 136 accessions of *Brassica* napus showing four categories of pod angles (straight, inclined, flat and droop).


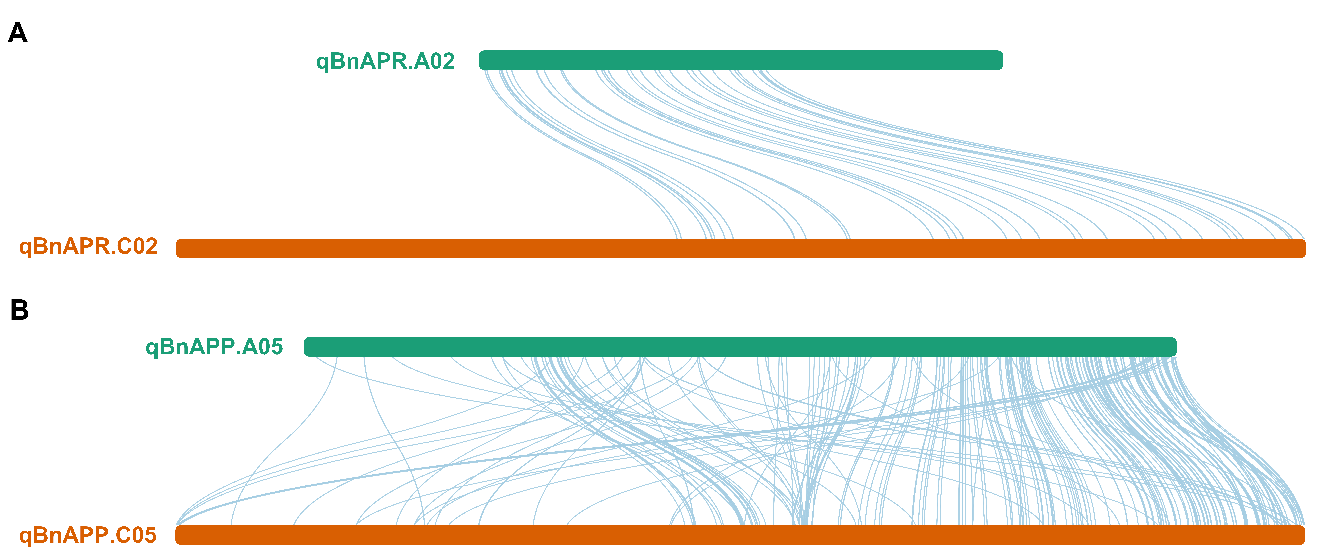


Fig. S5: Synteny analysis is to show that the QTL we are looking for were homologous. We used the genome of ZS11 for synteny analysis. (A) Synteny analysis of regional homology between qBnAPR.A02 and qBnAPR.C02; (B) Synteny analysis qBnAPP.A05 and qBnAPP.C05.


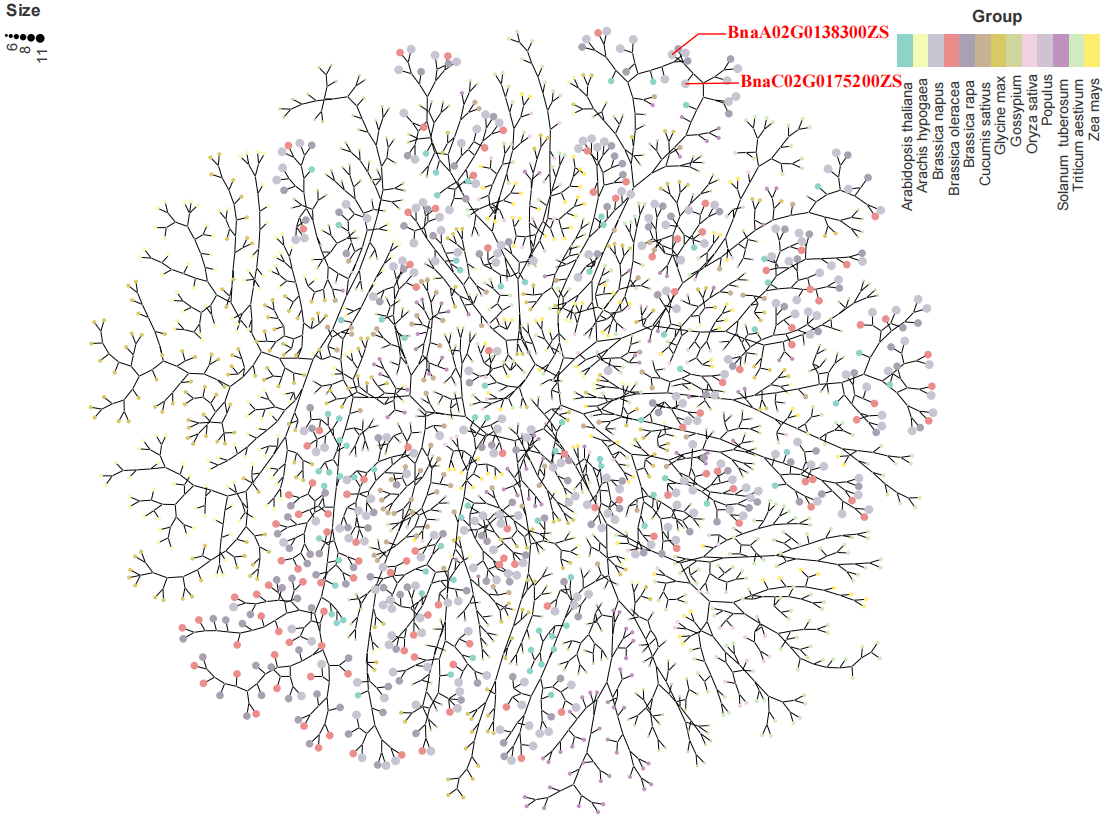


Fig. S6: Phylogenetic tree of SAUR gene family in 13 species, including *Arabidopsis thaliana, Arachis hypogaea, Brassica oleracea, Brassica napus, Brassica rapa, Cucumis sativus, Glycine max, Gossypium, Oryza sativa, Populus, Solanum tuberosum, Triticum aestivum and Zea mays*. Different colours represent different species, the size of the circle represents the genetic relationship with rape, and the larger the circle, the closer it is to rape.
